# The effects of doxorubicin loaded aptamer S3-linked DNA tetrahedrons on nasopharyngeal carcinoma

**DOI:** 10.1186/s40463-023-00673-2

**Published:** 2023-12-12

**Authors:** Xiwu Liu, Bincan Jiang, Ailan Cheng, Youwei Guo, Lei Wang, Weidong Liu, Wen Yin, Yihan Li, Xingjun Jiang, Caiping Ren

**Affiliations:** 1 NHC Key Laboratory of Carcinogenesis, Department of Neurosurgery, Xiangya Hospital, Central South University, Changsha, Hunan 410008 China; 2 National Clinical Research Center for Geriatric Disorders, Xiangya Hospital, Central South University, Changsha, Hunan 410008 China; 3 Cancer Research Institute of Hengyang Medical School, University of South China, Hengyang, Hunan 421001 China; 4 Cancer Research Institute, School of Basic Medical Science, Central South University, Changsha, Hunan 410078 China; 5 Department of Neurosurgery, Xiangya Hospital, Central South University, Changsha, Hunan 410008 China

**Keywords:** Nasopharyngeal carcinoma, Aptamer S3, CD109, 5-8F cells, DNA tetrahedrons, Doxorubicin

## Abstract

**Objective:**

Our research group in the early stage identified CD109 as the target of aptamer S3 in nasopharyngeal carcinoma (NPC). This study was to use S3 to connect DNA tetrahedron (DT) and load doxorubicin (Dox) onto DT to develop a targeted delivery system, and explore whether S3-DT-Dox can achieve targeted therapy for NPC.

**Methods:**

Aptamer S3-conjugated DT was synthesized and loaded with Dox. The effects of S3-DT-Dox on NPC cells were investigated with laser confocal microscopy, flow cytometry, and MTS assays. A nude mouse tumor model was established from NPC 5-8F cells, and the in vivo anti-tumor activity of S3-DT-Dox was examined by using fluorescent probe labeling and hematoxylin–eosin staining.

**Results:**

The synthesized S3-DT had high purity and stability. S3-DT specifically recognized 5-8F cells and NPC tissues in vitro. When the ratio of S3-DT to Dox was 1:20, S3-DT had the best Dox loading efficiency. The drug release rate reached the maximum (0.402 ± 0.029) at 48 h after S3-DT-Dox was prepared and mixed with PBS. S3-DT did not affect Dox toxicity to 5-8F cells, but reduced Dox toxicity to non-target cells. Meanwhile, S3-DT-Dox was able to specifically target the transplanted tumors and inhibit their growth in nude mice, with minor damage to normal tissues.

**Conclusion:**

Our study highlights the ability and safety of S3-DT-Dox to target NPC cells and inhibit the development NPC.

**Graphical abstract:**

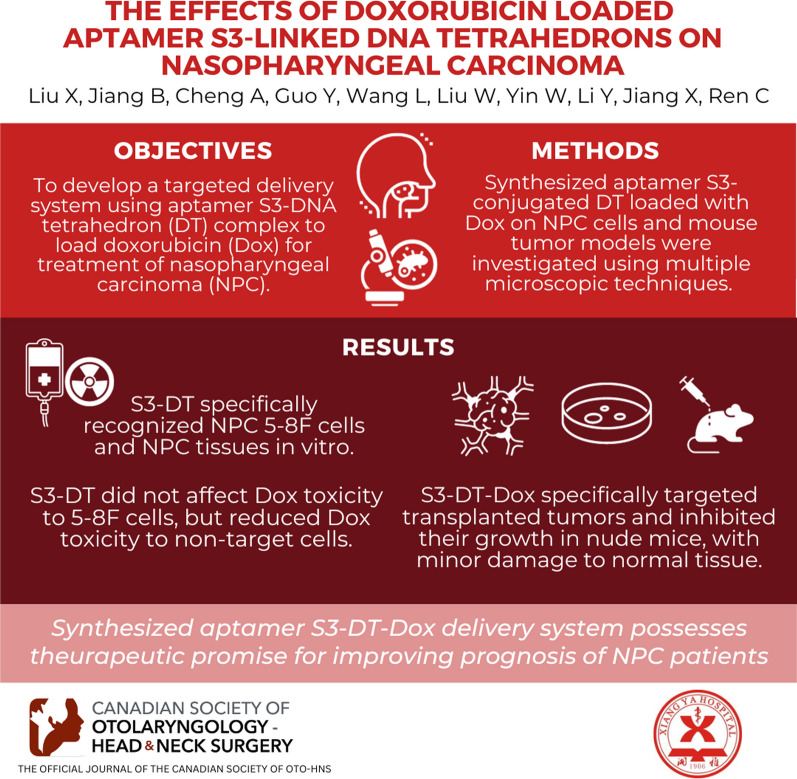

## Introduction

Nasopharyngeal carcinoma (NPC) is an epithelial malignant tumor originated from the uppermost part of the pharynx, covering the surface of the soft palate and the base of the skull [1]. Based on the epidemiological data for 36 cancers in 185 countries in the 2020 GLOBOCAN database, it is estimated that there are more than 133,000 new cases of NPC and about 80,000 deaths caused by NPC [2]. This malignancy is particularly prevalent in southern China, southeast Asia, northern Africa, and middle East regions, showing significant ethnic and geographic distribution characteristics [3]. Epstein-Barr virus (EBV) infection is regarded as a potential aetiological factor for NPC, which has been most extensively discussed [4]. In addition to EBV infection, the pathogenesis of NPC is also associated with genetic susceptibility and chemical carcinogens [5]. The past few decades have witnessed the research progresses in the genomic, epigenomic, and immune landscapes of NPC, which provide a theoretical grounding for the development of new biomarkers and therapeutic targets of NPC [6]. Concurrent chemoradiotherapy is the mainstay treatment for locoregionally advanced NPC, but acute and late toxicities (the latter may appear months or even years after the end of treatment) may be inevitable owing to high-dose radiation or chemoradiotherapy [7]. Doxorubicin (Dox) is one of the most frequently prescribed and time-tested anti-tumor agents. The combination of ifosfamide and doxorubicin is effective for treating recurrent and metastatic NPC patients [8]. But it still faces two major challenges as an anti-cancer chemotherapy, namely drug-caused cardiotoxicity and drug resistance [9]. Hence, new approaches that enhance the efficacy of Dox and overcome the toxicity and resistance are urgently needed.

Modifications of anti-tumor drug delivery systems have attracted great attention as they may help to deliver chemotherapeutic agents specifically to tumor cells, thus avoiding dose-related toxicity and off-target toxicity [10]. For instance, folate-conjugated polyethylene glycol (PEG)-phosphatidylethanolamine (PE)-modified poly(lactic-co-glycolic acid) (PLGA) nanoparticles have been proposed to be a promising drug delivery system for Dox and can act comprehensively to enhance the anti-tumor efficacy [11]. Aptamers, a class of versatile nucleic acid-based macromolecules presenting high affinity and specificity for their targets, show great potential as targeting moieties for nanocarriers to specifically recognize selective cancer cells and transfer anti-tumor drugs to target cells [12], expecting to increase therapeutic efficiency owing to multivalent effects [13]. A recent study has illustrated that a chimera consisting of two aptamers can be applied to deliver Dox to cancer cells, which improves the therapeutic efficacy and reduces the off-target cytotoxicity of Dox [14]. DNA tetrahedrons (DTs) are a kind of nanostructure formed from four short single-stranded DNA, which can be used as carriers for multiple anti-tumor agents such as floxuridine, camptothecin, and platinum drugs and deliver them to tumor cells if appropriately designed [15]. Several studies have shown that the widely-used anti-tumor drug Dox can be can be inserted between DNA base pairs and be effectively delivered to drug-resistant breast cancer cells by DT with or without aptamer modification [16, 17]. Aptamer-guided DTs with Dox increased cytotoxicity to PTK7-positive cells, with a minor effect on normal cells, showing potential application for T-cell acute lymphoblastic leukemia [18]. Yet few studies focus on this delivery system in NPC.

Our research group in the early stage identified CD109 as the target of aptamer S3 and a potential NPC biomarker [19]. Herein, we developed a delivery system in which Dox was loaded into CD109-recognizing aptamer S3-conjugated DT (S3-DT-Dox) and demonstrated its effect on NPC.

## Materials and methods

### Ethics statement

The animal experiments were implemented with the approval of the animal care committee of Xiangya Hospital of Central South University and complied with the rules and regulations and the operating norms of the management of experimental animals and the relevant ethical requirements. The experiments involving clinical samples were approved by the ethics committee of Xiangya Hospital of Central South University. Each individual who provided clinical samples signed an informed consent form.

### Cell culture

The human NPC cell line 5-8F (BeNa, Beijing, China) was grown in RPMI-1640 medium (Gibco, Grand Island, NY, USA) containing 10% fetal bovine serum (FBS) and supplemented with 1% penicillin/streptomycin, and the human normal nasopharyngeal epithelial cell line NP69 (ATCC, Manassas, Virginia, USA) was cultured in keratinocyte serum-free medium (KSFM) under the conditions of 37 °C and 5% CO_2_.

### Aptamer

The aptamer used in this experiment was S3 targeting the CD109 protein on the surface of the 5-8F cells, which was derived from the screening of NPC specific aptamers by Wenting Jia in the same laboratory. The aptamer S3 was screened with NPC cells as positive targets from synthetic DNA libraries by Cell-based Systematic Evolution of Ligands by Exponential enrichment and verified to be the most specific one among the selected sequences.

### Human sample collection

NPC tissues were taken from the tissue samples stored in Xiangya hospital, Central South University. The enrolled patients were NPC patients who visited the Otolaryngological Department of Xiangya hospital and underwent biopsy. All the nasopharyngitis tissues were resected from nasopharyngitis patients during biopsy. 

### Synthesis of DT, S3-DT, DT-Cy5, S3-DT-Cy5, DT-Dox, and S3-DT-Dox

The base sequences of four single-stranded DNA in DT are listed in Table 1.

**Table 1:** Base sequences of four single-stranded DNA in DNA tetrahedron

Number	Sequence
Strand 1 (A)	CGTATCACCAGGCAGTTGAGACGAACATTCCTAAGTCTGAAATTTATCACCCGCCATAGTAG
Strand 2 (B)	CGATTACAGCTTGCTACACGATTCAGACTTAGGAATGTTCGACATGCGAGGGTCCAATACCG
Strand 3 (C)	CGTGTAGCAAGCTGTAATCGACGGGAAGAGCATGCCCATCCACTACTATGGCGGGTGATAAA
Strand 4 (D)	CTCAACTGCCTGGTGATACGAGGATGGGCATGCTCTTCCCGACGGTATTGGACCCTCGCATG

For synthesis of DT, 10 μL of A, B, C, and D single-stranded DNA fragments (all at a concentration of 10 μM) were annealed at 95 °C for 10 min in 10 × TAE buffer (10 μL) and ultrapure water (50 μL), followed by natural cooling and storing at room temperature. DT with a final concentration of 1 μM was obtained.

For synthesis of S3-DT, 10 μL of A, B, C, and D single-stranded DNA fragments and S3 fragments (all at a concentration of 10 μM) were annealed at 95 °C for 10 min in 10 × TAE (10 μL) and ultrapure water (40 μL), followed by natural cooling and storing at room temperature. S3-DT with a final concentration of 1 μM was obtained.

For synthesis of DT-Cy5, 10 μL of A, B, C, and D-Cy5 fragments (all at a concentration of 10 μM) were annealed at 95 °C for 10 min in 10 × TAE buffer (10 μL) and ultrapure water (50 μL), followed by natural cooling and storing at room temperature. DT-Cy5 with a final concentration of 1 μM was obtained.

For synthesis of S3-DT-Cy5, 10 μL of A, B, C, and D-Cy5 fragments and S3 DNA fragments (all at a concentration of 10 μM) were annealed at 95 °C for 10 min in 10 × TAE buffer (10 μL) and ultrapure water (40 μL), followed by natural cooling and storing at room temperature. S3-DT-Cy5 with a final concentration of 1 μM was obtained.

For synthesis of DT-Dox, 50 μL of A, B, C, and D fragments (all at a concentration of 400 μM) were annealed at 95 °C for 10 min in 10 × TAE buffer (40 μL) and ultrapure water (160 μL, with a final volume of 400 μL and a final concentration of 50 μM), followed by natural cooling and incubation with 4 μL of 100 mM Dox for 3 h at 37 °C to obtain DT-Dox solution (DT 50 μM, Dox 1 mM), in which the amount of Dox was 0.233 mg.

For synthesis of S3-DT-Dox, 50 μL of A, B, C, and D fragments and S3 fragments (all at a concentration of 400 μM) were annealed at 95 °C for 10 min in 1 × TAE buffer (40 μL) and ultrapure water (110 μL, with a final volume of 400 μL and a final concentration of 50 μM), followed by natural cooling and incubation with 4 μL of 100 mM Dox for 3 h at 37 °C to obtain S3-DT-Dox solution (S3-DT 50 μM, Dox 1 mM), in which the amount of Dox was 0.233 mg.

### Agarose gel electrophoresis

2.5% agarose gel was prepared, and the electrophoresis buffer was added into an electrophoresis tank. Next, the prepared gel was placed in the center of the electrophoresis tank, and 50 bp of DNA Ladder, as the reference Marker, was added into the sample well of the gel. A chain, A + B chain, A + B + C chain, DT, and S3-DT were respectively mixed with the loading buffer and added to the sample wells. After that, the lid of the electrophoresis tank was closed, followed by turning on the electrophoresis device. The electrophoresis parameters were set as follows: voltage 120 V, current 400 mA, and electrophoresis time 30 min.

### Particle-size analysis

The particle size was measured using a Malvern particle size meter. Briefly, 1 pmol of S3-DT or DT was dissolved in 1 mL of double distilled water and placed in quartz plates for measuring the particle size.

### Stability detection of DT and S3-DT

At 37 °C without light exposure, 20 μL of DT or S3-DT (a final concentration of 1 μM) was incubated in 20 μL of McCoy'5A complete medium for 0, 2, 4, 6, 8, 10, 12, and 24 h, and the samples were taken at each time point and then stored at − 20 °C. The collected specimens were subjected to gel electrophoresis to check the bands. Next, 20 μL of DT and S3-DT solutions (a final concentration of 1 μM) were respectively mixed with 20 μL of FBS in a ratio of 1:1 and then incubated at 37 °C for 1, 3, 5, and 7 h. Specimens were taken at the time points and stored at − 20 °C for uniform gel electrophoresis.

### Laser confocal analysis

5-8F or NP69 cells (1 × 10^4^) were seeded and incubated in optical dishes for 24 h. Next, cells were washed three times with Dulbecco phosphate-buffered saline (DPBS), and incubated in binding buffer with the addition of Cy5-labeled S3, S3-DT, or DT with a final concentration of 250 nM at 37 °C for 3 h, followed by three washes in DPBS and imaging under a laser confocal microscope (Leica). Photographs were taken at a 63 × lens, with an excitation wavelength of 633 nm and an emission wavelength of 650–750 nm.

### Flow cytometry

For analysis of the ability of S3-DT to recognize NPC cells, 5-8F or NP69 cells (4 × 10^5^) were incubated in 12-well plates for 24 h. Cells were rinsed three times with DPBS before the experiment. The binding buffer with Cy5-labeled S3, S3-DT, or DT (250 nM) was added to 5-8F and NP69 cells and incubated at 37 °C for 3 h. After that, cells were washed three times with DPBS again and suspended in 400 μL of DPBS. Cy5 fluorescence in the cells was detected by flow cytometry.

For analysis of the uptake of the synthetic S3-DT-Dox by NPC cells, 5-8F or NP69 cells were scraped down from the culture flasks and washed two times with Hanks buffer. Subsequently, cells were incubated for 1.5 h with Dox, DT-Dox, or S3-DT-Dox at a Dox concentration of 2 μM at 37 °C. Next, the drug-treated cells were removed, and the PBS buffer containing experimental drugs was aspirated. The cells were washed three times with Hanks buffer, added to medium, and continued for 48 h-incubation at 37 °C. The cells were then rinsed two times with Hanks buffer, fixed with 4% formaldehyde for 10 min, and analyzed by flow cytometry.

### Fluorescence detection of NPC tissues

Paraffin sections of NPC tissues and nasopharyngitis tissues were heated for 1 h in a 60 °C oven to fully melt the paraffin, followed by dewaxing and hydration, and antigen repair. Then the sections were immersed in antigen repair solution, repaired under high pressure for 2.5 min, and naturally cooled to room temperature, followed by 3 times of washing with PBS (3 min each). Subsequently, the sections were blocked for 60 min at room temperature using aptamer blocking solution, and then cultured at 37 °C for 2 h with biotin-modified S3, S3-DT, or DT at a final concentration of 1 μM. Streptoavidin-modified ZnCdSe/ZnS quantum dot (QD) fluorescent nanoparticles were added and incubated at room temperature for 30 min. ZnCdSe/ZnS quantum dots were purchased from Wuhan Jiayuan QuantumDot Technology (Wuhan, Hubei, China). The solvent was 50 mM Borate buffer solution, pH = 8.4, 0.05% NaN_3_, concentration 1.0 μM, diluted at 1:100 during use, with excitation wavelength of 488 nm and emission wavelength of 605 nm. After 3 times of washing with PBS (5 min each), the sections were sealed with an anti-fluorescence quencher and photographed under a fluorescence microscope with an excitation wavelength of 488 nm and an emission wavelength of 600 nm.

### Optimum loading ratio of Dox in DT

When Dox was intercalated into DNA, its fluorescence would be quenched, which can be used to identify the efficiency of Dox intercalation into DNA structure. Briefly, 50 μL of 2 μM Dox (in TAE) was separately added with 0, 0.1, 0.5, 1, 2, 5, and 10 μL of S3-DT or DT (at a concentration of 1 μM), and added to 100 μL using 1 × TAE), so that the final concentration of S3-DT or DT was 0 nM, 1 nM, 5 nM, 10 nM, 20 nM, 50 nM, or 100 nM. The samples were incubated at 37 °C for 3 h, and the fluorescence spectrum of Dox was measured with a fluorometer, with an excitation wavelength of 480 nm and an emission wavelength of 540–700 nm. The drug loading curves of different DNA nanostructures at different molar ratios were plotted. Finally, the optimal drug loading ratio was obtained.

### Release efficiency of Dox

10 μL of S3-DT (1 μM), 50 μL of Dox (4 μM), and 10 μL of 1 × TAE (pH = 5) (a total volume of 100 μL) were incubated at 37 °C for 3 h to prepare S3-DT-Dox complexes (100 nM S3-DT and 2 μM Dox). The above solution was mixed with 100 μL of PBS, and then centrifuged (8000 rpm, 3 min) in a 3 K ultrafiltration tube. The filtrate containing the released Dox was obtained at various time points (0.5, 1, 2, 4, 8, 12, 24, 36, and 48 h). The fluorescence intensity of Dox in the filtrate was measured by a fluorometer, and the filtrate was put back into the ultrafiltration tube after the measurement. The drug release rate was calculated by the released Dox/total Dox (the fluorescence intensity of the filtrate after ultrafiltration of 1 μM Dox without S3-DT).

### 3-(4,5-Dimethylthiazol-2-yl)-5-(3-carboxymethoxyphenyl)-2-(4-sulfophenyl)-2H-tetrazolium (MTS) cell-viability assay

5-8F cells and NP69 cells in the logarithmic growth phase were trypsinized. After the cells were counted, cell suspension was made with a complete medium, and 5 × 10^3^ cells were seeded in each well of a 96-well plate and then cultured at 37 °C for 24 h. Adherent cells were washed two times with Hank’s buffer, followed by addition of an appropriate amount of PBS. Free Dox, DT-Dox, and S3-DT-Dox were added to the wells, and the final concentrations of Dox in each well were 0.01, 0.02, 0.2, 1, 2, 5, 10, 20, and 40 μM, respectively. Drug-treated cells were incubated at 37 °C for 2 h, washed three times with Hank’s buffer, and then cultured with medium containing 1% serum at 37 °C for 48 h. After that, the survival of cells in the 96-well plate was observed with an inverted microscope. After the removal of the culture medium, the cells were rinsed two times with Hank’s buffer. Next, 100 μL of serum-free medium was added to each well of the 96-well plate, followed by addition of 20 μL of the prepared MTS reagent (thawed MTS and PMS mixed at a ratio of 20:1). Light should be avoided during the above process. The 96-well plate supplemented with MTS reagent was incubated at 37 °C for 4 h, and the absorbance was measured at a wavelength of 490 nm with a microplate reader. According to the measured absorbance value, the cell viability was calculated, and the survival curve was plotted.

### Western blot

Cells were lysed using radioimmunoprecipitation assay lysis buffer (Beyotime, Shanghai, China) to obtain protein samples. After the protein concentration was measured with a BCA kit (Beyotime), the corresponding volume of proteins were mixed with the loading buffer (Beyotime) and heated in a boiling water bath for 3 min to denature the protein. Then, the protein samples were separated by 10% sodium dodecyl sulfate–polyacrylamide gel electrophoresis, transferred onto membranes (300 mA), and blocked in blocking solution at room temperature for 60 min or 4 °C overnight. Next, the membranes were incubated for 2–4 h with primary antibodies against CD109 (35717S, 1:1000, CST, Boston, MA, USA) and glyceraldehyde phosphate dehydrogenase (GAPDH) (5174S, 1:1000, CST), followed by 1 h-incubation with a secondary antibody. Lastly, the membranes were added with developer, and the detection was performed using a chemiluminescence imaging system (Bio-Rad, Hercules, CA, USA).

### Animal experiments

Forty-eight BALB/c nude mice (4–6 weeks old, 16 ± 2 g) of specific pathogen free (SPF) grade from the Laboratory Animal Resources of Chinese Academy of Sciences (Shanghai, China) were raised in a SPF grade sterile laminar flow chamber at constant temperature (25–28 °C) and constant humidity (about 50%). They were individually housed with free access to water and food under 12-h light/dark cycles. The padding was replaced every 3 days aseptically.

Fluorescence probe targeting and intra-organ metabolism experiments: 5-8F cells were injected into the armpit of the forelimbs of nude mice with 1 × 10^7^ cells per mouse, and the tumor was formed (5 mm × 4 mm × 4 mm) after 1–2 weeks for subsequent experiments. The nude mice were subjected to injection of 100 μL of DPBS, 100 μL of 50 μM S3-Cy5, 100 μL of 50 μM S3-DT-Cy5, or 100 μL of 50 μM DT-Cy5 via the tail vain, which were grouped as DPBS, S3, S3-DT or DT group, respectively (n = 6 mice/group). Subsequently, the above DNAs circulated in the mouse for 2 h, so that the aptamers were targeted to cancer tissues. The above mice were placed in a box containing isoflurane air for anesthesia. They were then placed in a small animal imager and imaged in the Cy5 fluorescence channel with the exposure time of 10 s. After that, the mice were dissected to obtain the organs, which were washed with DPBS, placed in petri dishes in sequence, and imaged with the small animal imager in the Cy5 fluorescence channel with the exposure time of 10 s.

For tumor xenografts analysis, 1 × 10^7^ tumor cells per mouse were injected into the armpit of the forelimbs of nude mice, and the subsequent experiments were performed when the tumors grew to 50 mm^3^. Four groups of tumor-bearing mice (DPBS, Free Dox, S3-DT-Dox, and DT-Dox, 6 mice for each group) were taken and respectively injected with 100 μL of DPBS, 100 μL of 1 mM free Dox, 100 μL of 50 μM S3-DT-Dox, or 100 μL of 50 μM DT-Dox (the amount of Dox was 3.5 mg/kg) through the tail vein every three days (1, 4, 7, and 10 days). From the first injection, the tumor volume was measured every two days for 14 days. On day 14, the mice were euthanized, and the tumors were isolated and weighed. Heart, liver, spleen, lung, and kidney tissues were taken and made into paraffin-embedded sections.

### Hematoxylin and eosin (HE) staining

The heart, liver, spleen, lung, and kidney tissues were fixed, dehydrated, and then cut into 5 μm thick slices with a microtome. Sections were routinely deparaffinized, stained with hematoxylin solution for 10 min, differentiated with 1% hydrochloric acid alcohol for several seconds, treated with 0.6% ammonia water for several seconds, counterstained with 0.5% eosin solution for 1–3 min, rinsed with tap water for 5 min, and rinsed with deionized water for 1 min, followed by routine dehydration, clearing, drying, and mounting. Finally, images were captured under a microscope (Olympus, Tokyo, Japan).

### Statistical analysis

Statistical analysis and graphing of data were performed using SPSS 18.0 (IBM Corp., Armonk, NY, USA) and GraphPad Prism 7.0. Data were presented as mean ± standard deviation. The comparison between two groups was performed using the *t* test, and the comparisons among multiple groups were conducted using one-way analysis of variance and Tukey’s post hoc test. A difference with *P* < 0.05 was considered to be statistically significant.

## Results

### CD109 expression in 5-8F and NP69 cells

**Fig. 1:**
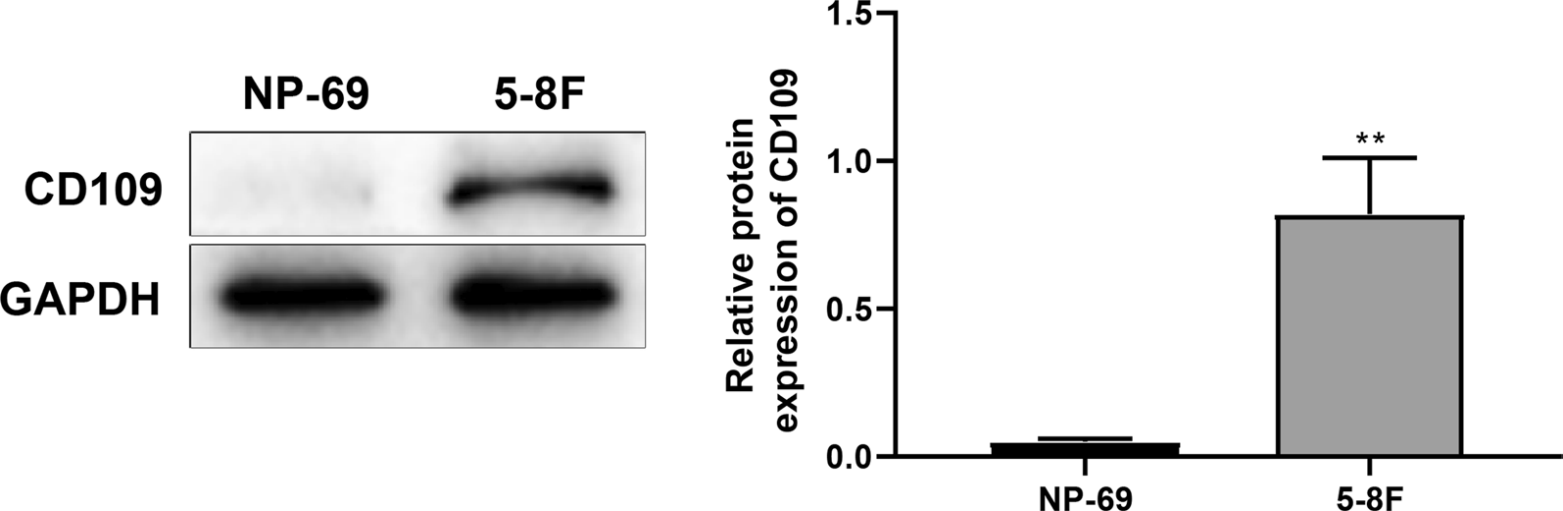
CD109 expression in 5-8F and NP69 cells. Western blot was performed to test CD109 protein expression in 5-8F cells and NP69 cells. The experiments were repeated thrice. **P < 0.01

Aptamer S3 specifically recognizes and binds to the CD109 antigen on the surface of 5-8F cells, but cannot recognize the control NP69 cells. Western blot was performed to test CD109 protein expression in 5-8F cells and NP69 cells, and the results revealed that CD109 protein expression could be detected in 5-8F cells but not in NP69 cells (Fig. 1).

### Synthesis and stability analysis of DT and S3-DT

**Fig. 2:**
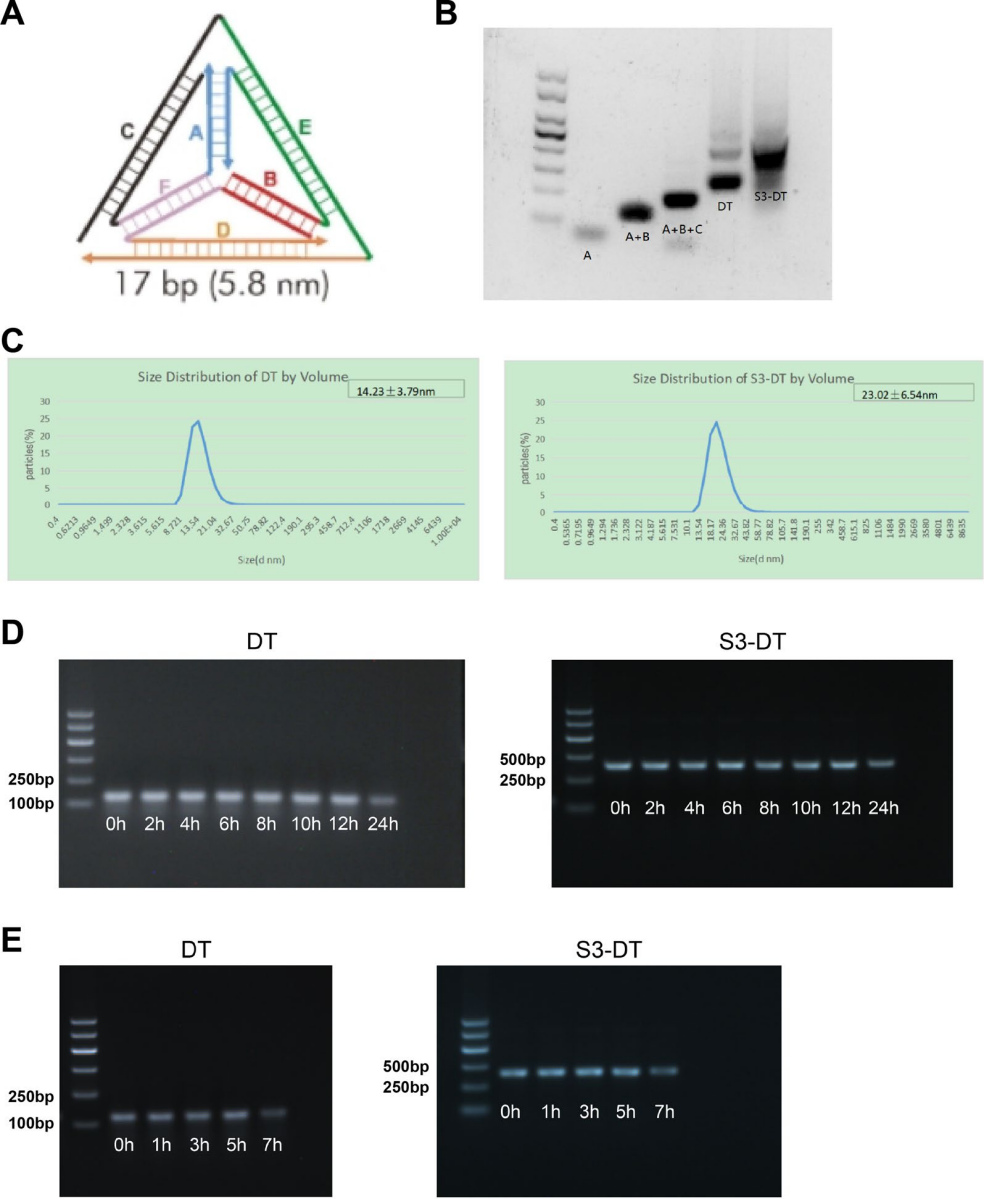
Synthesis and analysis of DT and S3-DT. **A** Four single-stranded DNA fragments formed a regular tetrahedral structure (pyramid shape) through complementary pairing. **B** Gel electrophoresis was utilized to detect the molecular sizes of A chain, A + B chain, A + B + C chain, DT, and S3-DT. **C** The particle size of DT was 14.23 ± 3.79 nm, and that of S3-DT was 23.02 ± 6.54 nm. **D** DT or S3-DT was incubated in a complete medium for 0–24 h and then detected by gel electrophoresis. **E** DT or S3-DT was incubated in 1:1 FBS for 0–7 h and then detected by gel electrophoresis. The experiments were repeated thrice

DT was synthesized from four single-stranded DNAs through complementary pairing (Fig. 2A). Because the A, B, C, and D chains all formed complementary pairs, the molecular weight of the complex would gradually increase with the increase in the chains. Therefore, we utilized gel electrophoresis to detect the molecular sizes of A chain, A + B chain, A + B + C chain, DT, and S3-DT. The results showed that the molecular size order was S3-DT > DT > A + B + C > A + B > A (Fig. 2B), indicating that we correctly synthesized DT and S3-DT which could be used for subsequent experiments.

Next, the particle sizes of DT and S3-DT were measured by a Malvern particle sizer (Fig. 2C). The particle size of DT was 14.23 ± 3.79 nm, PDI was 0.293 ± 0.05, and the particle size of S3-DT was 23.02 ± 6.54 nm, PDI was 0.378 ± 0.06; both of them had a single peak, indicating that the synthesized DT and S3-DT had high purity and good homogeneity.

In order to test the stability of DT and S3-DT, we incubated DT or S3-DT in a complete medium for different times and in 1:1 FBS for different times and then assessed the degradation of DT and S3-DT by gel electrophoresis. DT and S3-DT incubated in the complete medium for 12 h or less presented no significant degradation, and S3-DT with the 24-h incubation in the complete medium degraded to a certain extent (Fig. 2D). DT and S3-DT incubated in 1:1 FBS for 5 h or less showed no significant degradation, whereas there was slight degradation of S3-DT in 1:1 FBS upon 7 h incubation (Fig. 2E). DT stability was slightly reduced with the aptamer S3 ligation. Nonetheless, both DT and S3-DT showed good overall stability.

### Identification of NPC cells and tissues by S3-DT

**Fig. 3:**
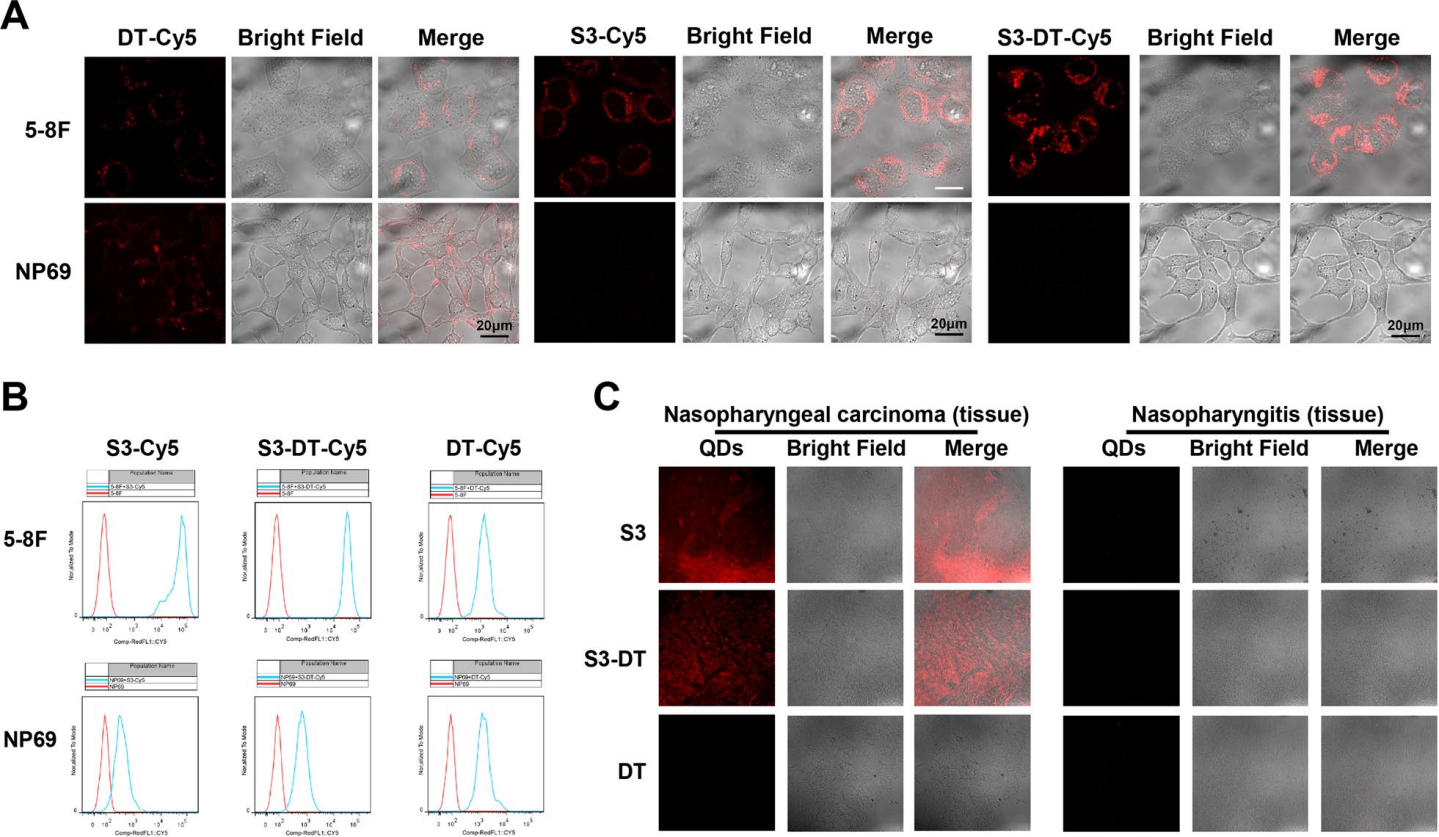
Identification of NPC cells and tissues by S3-DT. **A** Representative images from the laser confocal analysis when the Cy5-labeled DT (DT-Cy5), S3 (S3-Cy5), or S3-DT (S3-DT-Cy5) was incubated with 5-8F or NP69 cells for 3 h. **B** The fluorescence intensities of Cy5-labeled S3, DT, and S3-DT were tested by flow cytometry to analyze the ability of S3-DT to identify NPC cells. C. The recognition ability of S3-DT for NPC tissues was detected using fluorescence. The experiments were repeated thrice

We labeled DT, S3, and S3-DT with Cy5 to obtain DT-Cy5, S3-Cy5 and S3-DT-Cy5 respectively, which were then incubated with 5-8F or NP69 cells. Laser confocal microscopy was implemented to examine whether S3-DT could recognize and enter 5-8F or NP69 cells. Since DT had no ability to specifically recognize 5-8F and NP69 cells and differentiate between them, red fluorescence of DT-Cy5 could be observed in both 5-8F and NP69 cells. Since aptamer S3 had specific recognition of 5-8F cells, red fluorescence of S3-Cy5 could be observed only in 5-8F cells but not in NP69 cells. Meanwhile, S3-DT-Cy5 red fluorescence was observed only in 5-8F cells and not in NP69 cells (Fig. 3A). The above-mentioned results implied that the aptamer S3 could only recognize 5-8F cells, and that the connection of S3 to DT did not affect S3’s ability to specifically recognize 5-8F cells.

In addition, the ability of S3-DT to recognize NPC cells was analyzed by flow cytometry. The results showed that the fluorescence of DT-Cy5 was not different in NP69 and 5-8F cells and that the fluorescence intensity of S3-Cy5 and S3-DT-Cy5 in 5-8F cells was significantly higher than that in NP69 cells (red: blank control; blue: Cy5-labeled DNA incubated with cells) (Fig. 3B), indicating that DT-Cy5 has no specific ability to recognize 5-8F or NP69 cells. The blue fluorescence of S3-Cy5 and S3-DT-Cy5 in NP69 cells almost overlapped with the red fluorescence in blank control cells, indicating that neither S3-Cy5 nor S3-DT- Cy5 has the specific ability to recognize NP69 cells. While in 5-8F cells, the fluorescence of S3-Cy5 and S3-DT-Cy5 was stronger in comparison with that of DT-Cy5, indicating that both S3-Cy5 and S3-DT-Cy5 can specifically recognize 5-8F cells. Moreover, the similar Cy5 fluorescence intensities of S3-Cy5 and S3-DT-Cy5 reflected that there was no notable difference between S3-Cy5 and S3-DT-Cy5 in the ability to identify 5-8F cells. This result was consistent with the aforesaid laser confocal analysis, revealing that the aptamer S3 can specifically recognize 5-8F cells and that the connection of S3 to DT hardly affects the specific recognition ability of S3.

We further examined the ability of S3-DT to identify clinical NPC tissues and control nasopharyngitis tissues. In NPC tissues, red fluorescence was observed in the S3 and S3-DT groups but not in the DT group, while in nasopharyngitis tissues, no red fluorescence was observed in the S3, S3-DT, and DT groups (Fig. 3C). The results showed that both S3 and S3-DT could target NPC tissues but could not identify normal tissues, which provided the basis for subsequent targeted therapy.

### Effects of dox-loaded S3-DT on NPC cells

**Fig. 4:**
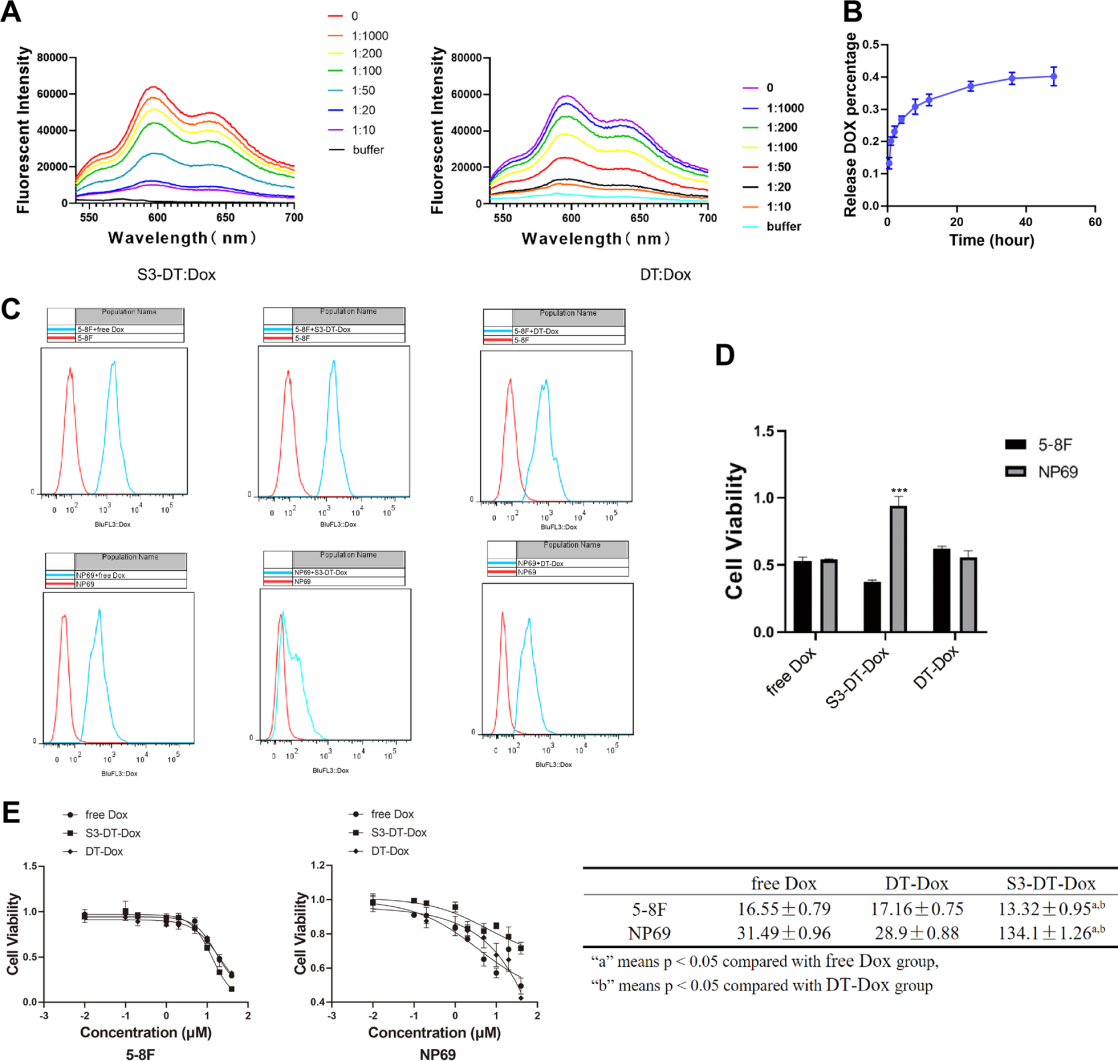
Effects of Dox-loaded S3-DT on NPC cells. **A** The optimal loading efficiency of Dox was tested using fluorescence. **B** The efficiency of Dox release from S3-DT was tested using fluorescence. **C** The uptake of S3-DT-Dox by NPC cells was determined by flow cytometry. **D**–**E** The toxicity (**D**) and IC50 values of S3-DT-Dox in 5-8F and NP69 cells (**E**) were determined by MTS assay. The experiments were repeated thrice. *P < 0.05, ***P < 0.001

The Dox fluorescence detection results suggested that the fluorescence intensity decreased with the increase of S3-DT or DT concentration (Fig. 4A), implying that with the increasing S3-DT or DT concentration, more and more Dox was loaded on S3-DT or DT. After reaching 50 nM, further increases in the concentration of S3-DT or DT could not reduce the Dox fluorescence intensity, indicating the optimal drug loading efficiency (by this time the ratio of S3-DT or DT to Dox was 1:20).

We then evaluated the efficiency of Dox release by examining the fluorescence intensity of Dox after its loading on S3-DT. The results showed that with the extension of time, the release of Dox was gradually elevated, and the maximum release rate was reached at 48 h (0.402 ± 0.029) (Fig. 4B).

After loading Dox on the S3-DT, we examined the uptake of the obtained S3-DT-Dox by NPC cells using flow cytometry. The results demonstrated that in NP69 cells, the Dox fluorescence intensity exhibited significant difference in the free Dox and DT-Dox groups when compared to that in the blank control group (Fig. 4C), suggesting that the Dox in the free Dox and DT-Dox groups was able to enter NP69 cells. However, the Dox fluorescence intensity of the S3-DT-Dox group overlapped with that of the blank control group, indicating that the loaded Dox could not enter NP69 cells because of the inability of S3 to recognize NP69 cells. In 5-8F cells, the Dox fluorescence intensities in the free Dox, DT-Dox, and S3-DT-Dox groups were significantly different in comparison to the blank control group, demonstrating that the Dox in all the three groups could enter 5-8F cells. The above results indicated that S3-DT-Dox specifically recognized 5-8F cells and delivered the loaded Dox into the cells. Meanwhile, S3 prevented the DT or DT-Dox from entering non-target cells and might reduce the damage to them.

Subsequently, the cytotoxicity of S3-DT-Dox to 5-8F and NP69 cells was evaluated by MTS assay. There were no significant differences in the toxicity of free Dox or DT-Dox to 5-8F and NP69 cells, but the toxicity of S3-DT-Dox to 5-8F was higher than its toxicity to NP69 (Fig. 4D). The IC50 values of free Dox and DT-Dox in 5-8F cells were not significantly different, but the IC50 value of S3-DT-Dox in 5-8F cells was greatly reduced. The IC50 value of S3-DT-Dox in NP69 cells was significantly higher than that of free Dox and DT-Dox (Fig. 4E). Taken together, the data indicated that S3-DT did not reduce the toxicity of Dox to 5-8F cells, but reduced the toxicity of Dox to non-target NP69 cells.

### Effects of S3-DT-Dox on growth of transplanted tumors and tissue damage in nude mice

**Fig. 5:**
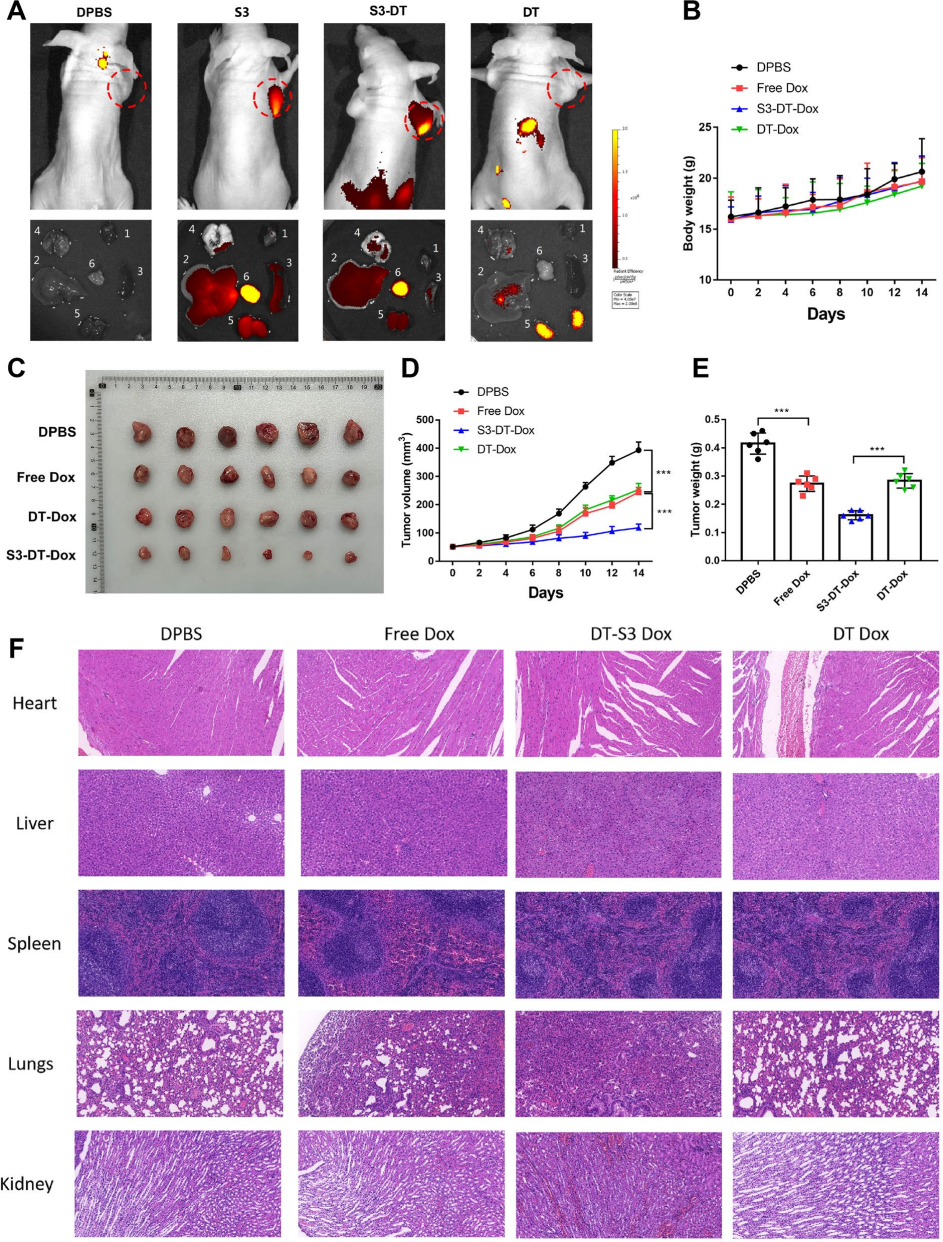
Effects of S3-DT-Dox on growth of transplanted tumors and tissue damage in nude mice. **A** The distribution of S3-DT in transplanted tumor nude mice was examined by in vivo imaging. **B** Changes in the body weight of nude mice during free Dox, DT-Dox, and S3-DT-Dox treatments. **C**–**E** Effects of free Dox, DT-Dox, and S3-DT-Dox treatments on the tumor growth in nude mice. **F** Tissue damage in the nude mice was determined by HE staining. n = 6. ***P < 0.001

We injected Cy5-labeled S3, DT, and S3-DT into nude mice with transplanted tumor through the tail vein to observe the distribution and tumor targeting of S3, DT, and S3-DT in mice by in vivo imaging. The results showed that no Cy5 fluorescence was observed in the DPBS group, no Cy5 fluorescence was observed in the tumor site of the DT group, and Cy5 fluorescence was observed in the transplanted tumors of both the S3 and S3-DT groups (Fig. 5A), revealing that aptamer S3 and S3-DT could target the transplanted tumor tissues. In addition, the fluorescence in the S3-DT group was more likely to accumulate in the tumor site of the nude mice, and the fluorescence aggregation in other tissues and organs was significantly reduced.

Next, we injected the Dox-loaded S3-DT into the nude mice through the tail vein to observe the effect of S3-DT-Dox on xenograft tumors in nude mice. We found that there were no significant change in the body weight and no difference in the weight among the groups (Fig. 5B, P > 0.05). Free Dox significantly inhibited tumor growth compared to DPBS (DPBS vs. free Dox, *P* < 0.001). The tumor inhibition of DT-Dox was comparable to that of free Dox (DPBS vs. DT Dox, *P* < 0.001; free Dox vs. DT Dox, *P* > 0.05). S3-DT-Dox, due to its tumor-targeting properties, had a better inhibitory effect on tumors than free Dox (S3-DT-Dox vs. free Dox, *P* < 0.001) and DT-Dox (S3-DT-Dox vs. DT Dox, *P* < 0.001) (Fig. 5C–E).

Finally, we examined the impact of S3-DT-Dox on the damage of other tissues (heart, liver, spleen, kidney, and lung tissues). Compared with free Dox and DT-Dox, S3-DT-Dox had less damage to normal tissues, especially the kidney (Fig. 5F). The above results suggest that S3-DT-Dox can achieve targeted therapy for NPC and reduce the toxicity to non-tumor tissues.

## Discussion

Advancements have been achieved in the management, radiotherapy, chemotherapy, and accurate tumor staging for NPC, contributing to the improvement of overall prognosis [20]. Among the chemotherapies, Dox has successfully treated recurrent metastatic NPC [21]. In this study, we set out to develop a delivery tool for Dox and our findings suggested that S3-DT-Dox could recognize target NPC cells and specifically deliver Dox to NPC cells to maintain its toxic effect on tumor cells without damaging normal cells. Furthermore, in vivo experiments evidenced that S3-DT-Dox not only targeted tumor grafts and exerted superior tumor-inhibitory activity compared to either free Dox or DT-Dox, but also attenuated the toxicity of Dox to other tissues.

Aptamers such as E3 aptamer have been extensively considered ideal for drug targeting due to their high-affinity and small size [22]. In a recent study, a modified aptamer-bound microfluidic chip shows a capture rate of ~ 90% in reorganizing NPC cells [23]. Our findings offered both in vitro and in vivo evidence suggesting the excellent ability of S3-DT-Dox to recognize NPC cells and inhibit NPC growth. Additionally, S3-DT-Dox exerted less toxicity to normal cells and organs. Multiple systems have been suggested as effective carriers of Dox for improvement of drug efficacy and attenuation of drug toxicity. A prior study has described a novel system consisting of PLGA-PEG-folate nanoparticles encapsulated with Dox, perfluorooctyl bromide (PFOB), and indocyanine green (ICG) as a targeted chemotherapeutic carrier traceable by either ^19^F magnetic resonance imaging (MRI) or near infrared (NIR) imaging, which holds great promise in diagnostic and therapeutic uses [24]. Also, the GE11-engineered graphene quantum dots (GQDs@GE11)/cisplatin (CDDP)/Dox nanoprobe shows a specific targeting effect that allows it to be recruited to the tumor site and further represses tumor cell proliferation [25]. More recently, another preliminary study has indicated that a 2-methoxyestradiol-emulsified drug delivery system can augment the cellular uptake of Dox by adriamycin-resistant breast cancer MCF-7 cells, showing promise to potentiate anti-tumor efficiency [26]. DT has also shown potential as a new nanocarrier system for improving the delivery of tumor-targeting drugs. An L-DNA tetrahedron is capable of targeting cancer cells and can strengthen the tumor accumulation of Dox and limit the Dox-induced cardiotoxicity [27]. DT holds great promise as an efficient carrier of Dox into drug-resistant breast cancer cells and a good candidate available for overcoming drug resistance of cancer cells [16]. Likewise, MUC1-targeting DT-conjugated AS1411 aptamer nanocarrier of Dox can reduce the drug cytotoxicity and resistance, whereby improving the drug efficacy in breast cancer [17]. Partially consistent with these findings, this study demonstrated that DT-Dox could also transfer Dox into 5-8F cells; however, only S3-DT-Dox specifically recognized 5-8F cells and did not affect the tumor-suppressive effect of Dox almost without damaging normal cells and tissues.

This study demonstrates that the prepared S3-DT specifically recognizes NPC cells, enhances the delivery of Dox, does not affect the toxicity of Dox on NPC cells, and reduces the toxicity of DOX on normal cells and organs. This modified delivery system possesses therapeutic promise for improving the prognosis of NPC patients.

## Data Availability

The datasets used or analyzed during the current study are available from the corresponding author on reasonable request.
